# Targeting the NOD-, LRR- and Pyrin Domain-Containing Protein 3 (NLRP3) Inflammasome in Psoriasis and Fatigue

**DOI:** 10.7759/cureus.24704

**Published:** 2022-05-03

**Authors:** Charmaine Kue Seguro, Michelle Demory Beckler, Marc M Kesselman

**Affiliations:** 1 Medicine, Nova Southeastern University Dr. Kiran C. Patel College of Osteopathic Medicine, Fort Lauderdale, USA; 2 Microbiology and Immunology, Nova Southeastern University Dr. Kiran C. Patel College of Allopathic Medicine, Fort Lauderdale, USA; 3 Rheumatology, Nova Southeastern University Dr. Kiran C. Patel College of Osteopathic Medicine, Fort Lauderdale, USA

**Keywords:** fatigue, cytokines, nlrp3 inflammasome, autoimmune, psoriasis

## Abstract

Inflammasomes are intracellular, multi-protein signaling complexes of the innate immune system that activate and control inflammatory responses in nucleated cells. Among these inflammasomes, the NOD-, LRR- and pyrin domain-containing protein 3 (NLRP3) inflammasome, a cytosolic sensor that modulates inflammatory responses in nucleated cells upon detection of various danger signals and microbial motifs, has been shown to a play a role in a wide range of pathologies and associated symptomatologies, including psoriasis and associated fatigue. Activation of the NLRP3 inflammasome can lead to caspase-1-dependent release of inflammatory cytokines, which potentially act on surrounding cells and may contribute to symptoms of fatigue. In this review, we will present recent developments in NLRP3 inflammasome research as it relates to psoriasis and fatigue, with a focus on the intracellular signaling pathways governing NLRP3 inflammasome regulation and promising pharmacological therapeutics that inhibit NLRP3 inflammasomal pathways.

## Introduction and background

Inflammasomes are intracellular, multi-protein signaling complexes of the innate immune system, which activate and control inflammatory responses in nucleated cells. Several types of inflammasomes are recognized, including NLRP1, NLRP3, and absent in melanoma 2 (AIM2), in the pathogenesis of psoriasis. Among these inflammasomes, NOD-, LRR- and pyrin domain-containing protein 3 (NLRP3) has been the most characterized and extensively studied [1,2] and will be the focus of this review. NLRP3 inflammasomes have been implicated in a wide range of pathologies and associated with symptomatologies [2], and are hyperactivated in individuals with autoimmune diseases, including psoriasis and corresponding fatigue [3].

Fatigue is a multi-dimensional, debilitating symptom commonly reported in high levels among 30% to 50% of patients with psoriasis and other inflammatory conditions. Fatigue is most commonly characterized by a significant sense of tiredness, lack of energy, and exhaustion [4]. Notably, patients with psoriasis place fatigue as having a significant impact on their quality of life [5,6]. Unfortunately, there is a paucity of efficacious long-lasting treatments of psoriasis and fatigue due to our limited understanding of the complex mechanisms surrounding its pathology [3].

Fatigue is commonly reported in many autoimmune disorders, and recent studies have described inflammation as a primary contributing factor in the pathophysiology of fatigue [7,8]. An interesting idea might be that there are inflammatory components responsible for its generation, as pro-inflammatory cytokines have been implicated in modulating various physiological functions contributing to fatigue, including stress [7], anxiety [8], depression [9], and sleep deprivation [10,11]. 

Dysregulation of inflammasome-mediated IL-1β and its receptors are implicated in fatigue in many autoinflammatory diseases [12,13]. Growing evidence also demonstrates the role of the NLRP3 inflammasome and its pro-inflammatory cytokines (e.g., IL-1β and tumor necrosis factor (TNF)-alpha) in patients with psoriasis [14-16], suggesting the potential of inhibiting NLRP3 and its cytokines in managing psoriasis and fatigue.

Below, we highlight recent reports that suggest a link between activation of NLRP3 inflammasome in psoriasis patients with possible consequent release of pro-inflammatory cytokines in these psoriasis patients. These pro-inflammatory cytokines regulated by NLRP3 inflammasome are also implicated in the underlying pathophysiology of fatigue. As such, the modulation of NLRP3 inflammasome in psoriasis patients may be responsible for patient-reported fatigue symptoms. Fatigue is a nebulous complaint with no measurable way to assess it and is rarely a treatment target. Thus, therapies targeting the NLRP3 inflammasome pathways may have significant potential in the management of fatigue and improvement in the quality of life in patients living with psoriasis.

NLRP3 inflammasome

**Figure 1:**
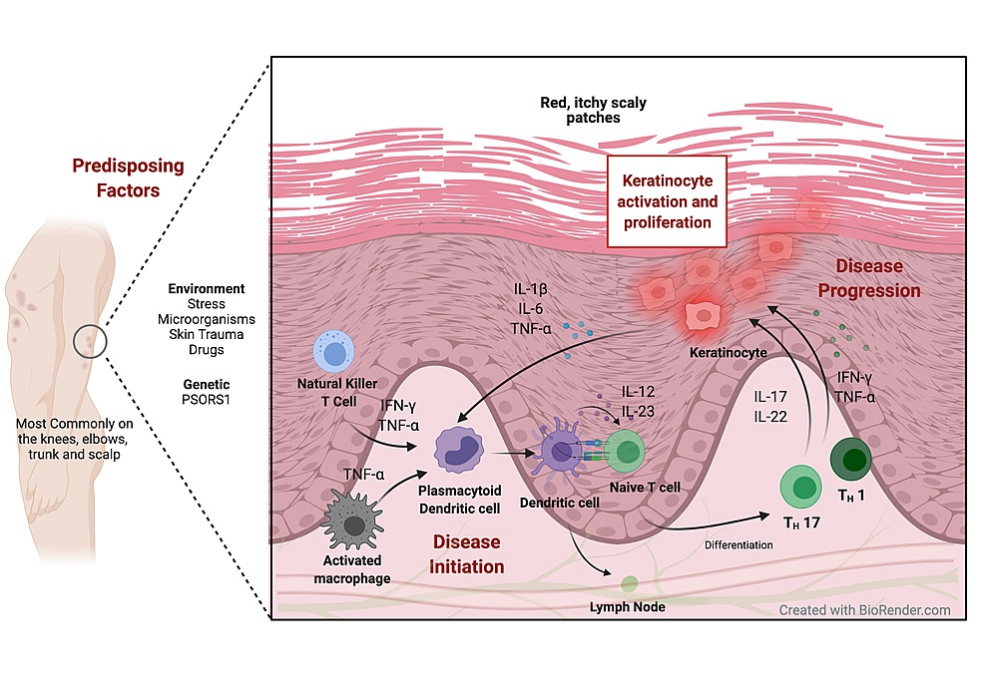
Proposed Schema of the Disease Progression in Psoriatic Lesions. Predisposing genetic (e.g., PSORS1 gene) and environmental factors (e.g., stress, skin trauma, or microbial infections) can trigger a cascade of events that lead to activation of innate immune cells, including plasmacytoid dendritic cells. Activated dendritic cells enter small lymph vessels and migrate into draining lymph nodes to induce differentiation of Naïve T cells into effector T cells, including Type 17 helper T cells (Th17) and Type 1 helper T cells (Th1). Production and release of pro-inflammatory cytokines are the key mediators for keratinocyte proliferation and subsequent appearance of skin lesions.

The onset of psoriasis is likely multi-factorial. Both genetically-susceptibility psoriasis susceptibility gene 1 (PSORS1) gene and environmental factors, including medications, stress, skin trauma, and infections (e.g., streptococcal and human immunodeficiency virus [HIV]), are implicated in disease onset [17]. These factors can lead to disruptions in innate and adaptive immunity, keratinocyte differentiation and proliferation, and the subsequent appearance of skin lesions [18,19] (Figure 1).

Meanwhile, the underlying immunopathogenesis of psoriasis remains insufficiently understood. It is thought that environmental factors contribute to the presence of pathogen-associated molecular patterns (PAMPs) and damage-associated molecular patterns (DAMPs) that bind to pathogen recognition receptors (PRRs) in immune cells and keratinocytes of the epidermis [20,21]. PAMPs alert the immune system to the presence of pathogenic infections derived from microorganisms. Conversely, DAMPs are host-derived cellular stress signals that are endogenously released in response to unscheduled cell death due to trauma, ischemia, or tissue damage [22].

**Figure 2:**
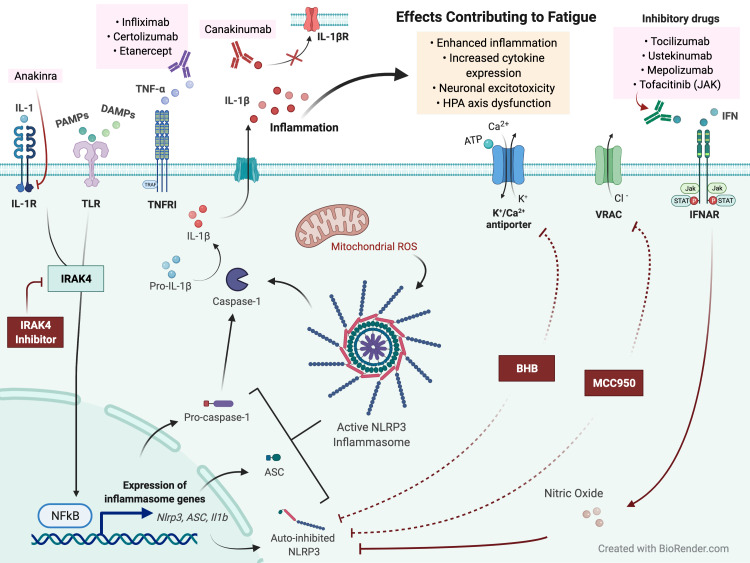
Proposed Schema of Activation and Inhibition of the NLRP3 Inflammasome. Attachment of PAMPs and/ or DAMPs (i.e., TNF-α or IL-1β) to their respective PRRs (i.e., tumor necrosis factor receptor 1 [TNFR1] and interleukin-1 receptor [IL-1R]) can activate transcriptional factors nuclear factor kappa B (NF-κB). Expression of inflammasomal genes primes components of the NLRP3 inflammasome, including NLRP3, apoptosis-associated speck-like protein containing a CARD (ASC), and pro-caspase-1. Metabolic alterations that induce reactive oxygen species (ROS) can act as a secondary signal to trigger the formation of the inflammasomal complex. The inflammasomal assembly then induces the cleavage and activation of procaspase-1 into caspase-1, which can proteolytically cleave the pro-forms of IL-1 family members into their mature, active forms. Upon release, these pro-inflammatory cytokines can alter surrounding cells which can lead to effects contributing to fatigue. NLRP3 inflammasomes could be inhibited by IL-1 receptor-associated kinase 4 (IRAK4) inhibitors to prevent NF­κB expression, and Type I interferons (IFNs) could inhibit NLRP3 through nitric oxide generation. β-hydroxybutyrate (BHB) and MCC950 presently have undefined mechanisms. BHB is purported to prevent K+ efflux, resulting in reduced oligomerization of NLRP3, and MCC950 may block the ATPase domain of NLRP3, mechanisms that could inhibit NLRP3 inflammasome activation.

NLRP3 Inflammasomes are cytosolic sensors activated by PRRs of cells, and attachment of PAMP and DAMP molecules to their respective PRRs can lead to activation of nuclear transcription factor-­κB (NF­κB) [23,24]. NF­κB primes the components of the NLRP3 inflammasome, including NLRP3, ASC, and Procaspase-1 [24]. Metabolic alterations that induce ROS can act as a secondary signal to trigger the formation of the inflammasomal complex. NLRP3 assemblies into a multi-protein complex, resulting in the cleavage and activation of procaspase-1 into caspase-1. Caspase-1 proteolytically cleaves inactive precursors of the interleukin-1 (IL-­1) family (e.g., IL-1β and IL-18) into mature, activated pro-inflammatory cytokines. These are subsequently released, causing immune-inflammatory reactions in surrounding cells that are purported to contribute to symptoms of fatigue [3,23] (Figure 2).

Role of NLRP3 inflammasome in psoriasis and fatigue

Keratinocyte release of IL-1 family cytokines and constitutive activation of the pro-inflammatory NF-κB pathway may be involved in the pathogenesis of psoriasis [25]. Most recently, research efforts have demonstrated that patients with psoriasis have increased expression of the NLRP3 inflammasome in plasma, along with caspase-1 reactivity and elevated inflammasome-generated IL-1β and IL-18 concentrations [16]. It has also been shown that inflammasome-generated IL-1β is associated with increased systemic production of IL-17 [26,27], a key cytokine that is profoundly implicated in plaque psoriasis. Thus, IL-1β is now emerging as a critical cytokine in the pathogenesis of IL-17-mediated skin disorders, and in particular, psoriasis [28].

Inflammasome-related genes and inhibitors of inflammasomes have also been recognized in the pathogenesis of psoriasis. For instance, Su et al. reported that the expression of NLRP3 in psoriatic samples was 3.5 to 4.3 times higher than the expression of NLRP3 in normal skin biopsy samples [29]. Irrera et al. also shows that BAY 11-7082, an antagonist of NF-κB, can alleviate psoriasis-like dermatitis in a mouse model by inhibiting the NLRP3 inflammasome and the NF-κB pathway [30].

Accumulating evidence indicates a link between inflammation and fatigue in clinical settings [31,32]. Specifically, dysregulation and increased production of IL-1β and its receptors are implicated in fatigue in various autoimmune diseases [12]. Elevation of IL-1 in the brain, for instance, contributes to “sickness behaviors” associated with fatigue, which includes depression, social anhedonia, and sedentary behavior [33].

Pro-inflammatory cytokines released by psoriatic keratinocytes or by systemic inflammation may reach the brain through several mechanistic pathways (e.g., humoral pathway via more permeable sections of the blood-brain barrier [BBB] or through a neuronal pathway via the vagus nerve [34-36].

In the humoral pathway, cytokines can cross more permeable areas of the BBB via diffusion through the fenestrated endothelium, especially those surrounding the circumventricular organs [37-39]. IL-1β and TNF-α can also translocate via a saturable transport system from blood to the central nervous system (CNS) [37]. In the neuronal pathway, cytokines can activate the vagus nerve or other afferent nerve fibers in the periphery [40], which then modulates relevant brain regions and functions [34]. Cytokine signaling and neuroinflammation can propagate via diffusion, direct neuronal projections, or microglial activation [36].

One mechanism by which inflammatory cytokines modulate neuronal function is through inhibition of dopamine and serotonin biosynthesis [41]. Recently, it has been suggested that these neurotransmitters can play a significant role in the pathophysiology of psoriasis [42]. Reductions in serotonin, for instance, may increase concentrations of inflammatory mediators such as IL-1β, TNF-α, and interferon-γ (IFN-γ), which can induce activation of nuclear factor-KB, keratinocyte proliferation, and worsening of psoriatic symptoms [43-45].

Targeting the pathways to treat psoriasis

Aberrant activation of the NLRP3 inflammasome has been implicated in a plethora of diseases, evoking a considerable amount of clinical interest in discovering potential inflammasomal targets for NLRP3 inhibition. Recent studies have highlighted potential pharmacological inhibitors in various sites of the NLRP3’s complex signaling cascade, including suppression of upstream signaling, prevention of inflammasomal assembly, and neutralization of the inflammatory cytokines that are released upon activation of the inflammasome [46-48].

In this review, we present several pharmacological interventions inhibiting the NLRP3 inflammasome complex (Table 1), their proposed mechanisms of action, key biological target sites, and purported therapeutic potential (Figure 2).

**Table 1:** Potential Interventions Against the NLRP3 Inflammasome. IL-1β: Interleukin 1 beta. BHB: β-hydroxybutyrate

Compound	Potential Mechanism of Action	References
*IL-1β Inhibitors*	Binds to IL-1 receptors, preventing attachment of IL-1β mediators.	[49-52]
MCC950	Inhibits NLRP3 activation via blocking its ATPase domain.	[53,54]
BHB	Prevents ASC oligomerization of NLRP3 via K+ efflux inhibition.	[55,56]

IL-1β

Growing evidence suggests that the brain acts as a central regulator of fatigue. Pro-inflammatory cytokines can play a key role in the development of severe fatigue via their inflammatory effects on the CNS [57]. Monoclonal antibodies targeting IL-12, IL-23, and IL-17 also show promise for the treatment of moderate to severe forms of psoriasis in clinical trials [58]. Notably, IL-1β is one of the most common and potent cytokines released via NLRP3 activation and has been implicated as the main regulator in numerous NLRP3-mediated conditions [31,59]. Currently, many pharmaceuticals are available that inhibit IL-1β, including canakinumab, a neutralizing IL-1β, anakinra, a recombinant IL-1 receptor antagonist, and rilonacept, a soluble decoy IL-1 receptor [1]. Thus, interleukin-1 (IL­1) inhibition presents an attractive option to treat NLRP3­dependent pathologies and associated fatigue [60].

Numerous studies demonstrate that inhibition of IL-1 signaling pathways can act as immunomodulatory therapies among patients with systemic inflammation, including severe acute respiratory syndrome coronavirus-2 (SARS-CoV-2) infection [61], atherosclerotic disease [59], diabetes [40], rheumatoid arthritis [62], and Sjogren syndrome [63]. In case reports, canakinumab and anakinra have been shown to be effective in the treatment of pustular psoriasis [50-52]. Anakinra has also been used in patients with plaque-type psoriasis and psoriatic arthritis [49] and was the first to be developed in treating other NLRP3­-associated diseases, including cryopyrin-associated periodic syndromes (CAPS), deficiency of IL-1 receptor antagonist (DIRA), and rheumatoid arthritis [64,65]. Although potentially effective in some patients with pustular psoriasis and psoriatic arthritis, it purportedly has limited therapeutic use in psoriasis due to the abundance of IL-1Ra in psoriatic lesions [66]. Anakinra also showed limited reductions in fatigue severity among female patients with myalgic encephalomyelitis/chronic fatigue syndrome (ME/CFS) in a four-week randomized, controlled trial [33], likely attributable to the presence of insufficient active concentrations in the cerebrospinal fluid that lacked a functional effect. Anakinra treatment has also been implicated in new-onset psoriasis [67] and is not commonly used due to its short plasma half-life of four to six hours, which would require the use of daily injections [68].

Canakinumab selectively blocks IL-1β mediators and neutralizes its function by preventing binding to IL-1 receptors, subsequently downregulating cellular signaling events that trigger IL-1β-driven gene activation and production of other inflammatory cytokines [69]. Canakinumab has been considered the preferred treatment option due to its longer half-life of 26 days (as compared to anakinra) [70] and has been shown to reduce the incidence of other inflammatory NLRP3-­related diseases, including pustular psoriasis and rheumatoid arthritis [71]. Meanwhile, it is important to note that activated NLRP3 inflammasomes do not only release IL-1β mediators but a host of cytokines that can also be mediated by other inflammasomes and other associated pathways [72]. Thus, pharmaceutical agents directly inhibiting the NLRP3 inflammasome might be a preferred approach in the management of NLRP3-based diseases.

MCC950

To date, MCC950 (also known as CRID3 and CP-456,773) originates from a family of sulfonylurea-containing compounds and is considered one of the most potent and selective NLRP3 inhibitors available for in vitro and in vivo experimentation [60]. Initially identified by Gabel et al., MCC950 acts by inhibiting IL-­1β release and caspase-1 processing in response to NLRP3 activation [54]. MCC950 shows high specificity to the NLRP3 inflammasome without inhibiting other inflammasome types or other toll-like receptors (TLR)-driven mechanisms. While the molecular target of MCC950 remains relatively undefined, MCC950 has been shown to inhibit NLRP3 activation by all known stimuli, highlighting that it is possible to produce an inhibitor that can directly inhibit the NLRP3 inflammasome and its activation pathways [60]. One study postulated that MCC950 functions by inhibiting chloride efflux after nigericin-induced stimulation of the NLRP3 inflammasome [53]. Another study demonstrated that gramicidin, a separate NLRP3 ­modulating ionophore, resulted in the same outcome through the influx of chloride ions [55]. As such, more research is needed to gain insight into the potential role of chloride channels in MCC950 regulation (Figure 1).

MCC950’s efficacy and therapeutic potential confer potential for use in a wide range of diseases, especially as research efforts provide compelling evidence for direct inhibition of NLRP3. Still, there is no standardized dosing of MCC950 exists for NLRP3 inhibition. In addition, studies evaluating MCC950’s effectiveness in rheumatoid arthritis were halted after phase II clinical trials due to liver injury, though it was surmised that the dose of 1,200 mg per day used in the study (in reaction to its furan moiety) likely led to drug-induced liver toxicity [1].

Diet and exercise

β-hydroxybutyrate (BHB), an endogenous ketone body produced in response to energy deprivation, has been shown to exert anti-inflammatory effects through NLRP3 inhibition [48]. BHB is produced in the liver and serves as an alternative energy source for the brain, heart, and other peripheral tissues when insufficient glucose stores are available for ATP production. Substantial NLRP3 inhibition occurs between two and four mM, and circulating BHB levels can rise to six to eight mM as glycogen stores in the liver are consumed in response to prolonged fasting, carbohydrate-restricted ketogenic diets (<50 g carbohydrates/day), and high-intensity exercise [73,74].

In one study, Youm et al. showed that BHB dose-dependently attenuates IL-1β secretion and adenosine triphosphate (ATP)-induced activation of caspase-1 in bone marrow-derived macrophages (BMDMs) in mice [48]. Mechanistically, it has also been suggested that BHB inhibits NLRP3 by blocking K+ efflux, decreasing intracellular potassium levels, and preventing oligomerization of ASC (apoptosis-associated speck-like protein containing a caspase recruitment domain) required for inflammasome activation [55,56]. BHB has also been shown to attenuate the processing of inflammatory cytokines in response to various PAMP molecules (e.g., Pam3CSK4, lipid A and lipotoxic fatty acids) [48]. In addition, BHB led to reductions in NLRP3-mediated IL-1β and IL-18 production in human monocytes. This inhibition occurred at similar elevated BHB concentrations induced by two days of fasting or high-intensity exercise [73,74].

Thomsen et al. suggested that patients with psoriatic arthritis can tolerate high-intensity interval training (HIIT), performed at 85% to 95% of HRmax, with significant improvements in fatigue after 11 weeks of training [75]. Aerobic exercise at 40% to 70% of HRmax has also been demonstrated to reduce fatigue in individuals with ME/CFS [76]. Studies in other inflammatory conditions, including rheumatoid arthritis and spondyloarthritis, have been shown to reduce inflammatory markers after HIIT training [77,78]. While HIIT has demonstrated no alterations in disease activity markers, many studies suggest that patients with psoriasis can attenuate feelings of fatigue by improving physical activity alone. It is possible that elevated serum endorphin concentrations induced by exercise and improvements in cardiorespiratory fitness could have led to reductions in fatigue [75]. Notably, improvements in fatigue may be temporary if the patient does not remain physically active. Thus, it is imperative for healthcare providers to prescribe exercise and make physical activity promotion a standard in clinical care.

Findings from Castaldo et al. also suggest that following a calorie-restricted, ketogenic diet (<500 kcal/day) can lead to psoriasis disease regression. Dermatology Life Quality Index (DLQI) was measured to determine the patient’s quality of life at baseline and four weeks, along with Psoriasis Area Severity Index (PASI), an index used to express the severity of psoriasis, and visual analog scale (VAS), a tool widely used to measure pain. Results show significantly reduced DLQI, PASI, and VAS pain after four weeks of treatment alone, suggesting that following a low-calorie ketogenic diet may be a viable treatment option for individuals with psoriasis. [79]. BHB-mediated inhibition of NLRP3 presents the possibility of treating inflammatory conditions without pharmaceutical use, suggesting that diet and lifestyle modifications, either through caloric restriction or vigorous exercise, can help treat NLRP3-mediated inflammatory diseases by increasing endogenous secretions of BHB.

## Review

Discussion

Based on this review, we found that inflammation is likely a potential contributing factor in the development and persistence of fatigue in patients with psoriasis [80]. This can impair individuals’ activities of daily living with direct consequences to their overall health and quality of life. Recent research reveals a relationship between inflammation and fatigue, and we have seen that this hypothesis is supported by clinical studies between fatigue and biomarkers of inflammation [81,82], including preclinical studies demonstrating symptoms of fatigue through exposure to inflammatory stimuli [83]. Further analysis implicates the involvement of NLRP3 inflammasomes via inflammatory cytokines in modulating physiological functions associated with fatigue [7-11,83].

Sex differences also exist in the prevalence of autoimmune diseases. Specifically, female patients are generally more affected than males, and not much is known regarding these biological differences [84]. Females, and in particular minority women, also remain underrepresented in clinical trials, limiting the development and adaptation of new therapeutics suitable for women [85]. A detailed understanding of the exact mechanisms of fatigue, including the development and severity of fatigue in patients with psoriasis, must be delineated. To ensure broader inclusivity, clinical trials focusing on gender and ethnic considerations should be a topic for future research. Trials targeting the NLRP3 inflammasome in these populations could also provide new insights into our understanding of the complex mechanisms surrounding this disease.

Interventions inhibiting the activation of the NLRP3 inflammasome may be effective in managing autoinflammatory diseases [60]. They demonstrate significant potential to alleviate symptoms of fatigue, thus improving the quality of life in patients with psoriasis. Potential pharmacological and non-pharmacological interventions exist to block various sites of the NLRP3’s signaling cascade, including inhibition of the NLRP3 complex's formation and cytokine neutralization, which would otherwise be released upon activation of the NLRP3 inflammasome [46-48,55]. Approved therapeutic interventions to induce long-term remission and attenuations in psoriasis and fatigue remain to be uncovered. Although the IL-23/ Th17/ IL-17 pathway has been established as the key major axis in psoriasis [86], there is also a subset of Western and Asian patients whose symptoms cannot be alleviated by pharmaceutical therapeutics targeting this pathway [87].

Future research efforts can make use of the availability of NLRP3 structure by focusing on the development of the structure-based design of pharmaceuticals with improved specificity and pharmacokinetic properties [1,88]. Although the complexity of the NLRP3 inflammasome is emerging within the context of psoriasis and fatigue, significant gaps in our understanding of the specific regulatory mechanisms governing the pathology of psoriasis and its association with the NLRP3 inflammasomes remain. Accumulating data from clinical and laboratory studies of NLRP3-associated biomarkers and novel pharmacological interventions that target NLRP3 pathways can provide promising insights for future research efforts and clinical practice management.

A patient who accounts for the ineffectiveness of pharmaceutical treatments to mitigate fatigue also underscores the potential for using conservative treatments [89]. It is also noteworthy to mention that many patients with psoriasis hold negative views on their management of fatigue, as they find it either dismissed or ignored in clinical settings [90]. Thus, focusing on conservative measures can potentially help clinicians design interventions to guide patients in the self-management of fatigue.

Increasing BHB, either through HIIT protocols or restrictive caloric diets alone, can lead to significant reductions in NLRP3-mediated IL-1β and IL-18 [73,74]. Patients with psoriatic arthritis can tolerate high-intensity interval training (HIIT) (85% to 95% of HRmax) with improvements in fatigue [75]. Findings also suggest that following a calorie-restricted, ketogenic diet (<500 kcal/day) can lead to psoriasis disease regression and improve a patient’s quality of life. Based on our evaluation of the current literature, we find that future investigations into BHB-mediated inhibition of NLRP3 can present the possibility of treating inflammatory conditions without pharmaceutical use and assist clinicians in designing interventions for patients. More importantly, improving patient awareness of the benefits of nutrition and exercise, using a progressive approach in clinical sessions, discussing barriers to change, and supporting patients to resolve these challenges can engage patients in the self-management of fatigue [91].

## Conclusions

NLRP3 is a central component of the innate immune system that underpins a broad spectrum of inflammatory disorders. Accumulating evidence demonstrates the role of NLRP3 in psoriasis patients, with potential reductions in disease activity and fatigue from mediating pro-inflammatory cytokines (e.g., IL-1β and TNF-alpha) regulated by the NLRP3 inflammasome. Additionally, patients with psoriasis may feel less fatigued following increased physical activity (e.g., HIIT), with no evidence to suggest that it may worsen outcomes. As such, modulating NLRP3 and associated pathways in psoriasis patients may have significant potential in the management of fatigue and improvement of life in patients with this condition. As the number of individuals with severe inflammatory conditions driven by the NLRP3 inflammasome increases over time, there is a strong impetus for the development of therapeutic compounds and conservative treatments that selectively inhibit NLRP3. Hence, it necessitates investigating how these interventions can impact the specific pathophysiological pathways of fatigue.
